# Removal of Both Ovaries, with Report of a Case

**Published:** 1883-07

**Authors:** J. H. Etheridge

**Affiliations:** Chicago


### Original Communications.

### ARTICLE II.

REMOVAL OF BOTH OVARIES, with Report of a Case. By J. H. ETHERIDGE, M.D., Chicago. (Read before the Chicago Medical Society, September 16, 1882.)

"Battey's operation," or "oöphorectomy," or "normal ovariotomy," as the removal of the ovaries, or castration of women, is now termed, is one of the comparatively new operative procedures. As it promises to accomplish in relieving certain troubles more than any known method of treatment, it becomes an interesting and profitable field of study and observation.

The following report is of a case where oöphorectomy was the only untried means of relief. The patient, of a nervous, sanguine temperament, aged twenty-five years, had suffered for years from a dysmenorrhœa whose severity is seldom equalled.

In childhood she had measles twice. Nothing else bearing on her present condition seems to be worthy of mention till her twelfth or thirteenth year, when she began to suffer from hyperæsthesia over the ovarian and uterine regions, and has never known what it is to be free from it since.

Till her sixteenth year she was in and out of school, always overworking in study, thereby being compelled to remain out for a time.

She commenced to menstruate at fourteen and one-half years of age, and always flowed freely; suffered severely with each period from the first. Till her sixteenth birthday she was irregular, when she began to suffer intensely from a burning pain through the right ovarian region, hip and leg, accompanied by tenderness over the right ovary. Very soon thereafter, in February, 1875, she was thrown from a sleigh, and sustained a severe shock, from the effects of which she did not rally for six months. Her disorder then was pronounced "concussion of the brain." Since that accident she has never been as well. For many months thereafter, she was delirious for a day and night with each menstrual period, and when at last sleep came, it was disturbed by her frequently starting up and crying out to be saved from falling. During three months she had constant cephalalgia to such an extent that she could not read nor write. Since then her neuralgia has increased. Whenever the fresh cerebral congestion incident to menstruation has been present, she has had general, as well as, at times, localized hyperæsthesia very severely. As the years have rolled by, this hyperæsthetic condition increased, till her general condition of suffering at menstruation was well-nigh beyond the power of words to describe.

After the accident, her dysmenorrhœa grew progressively worse in every particular, till in the past seven years she presented a condition of suffering, neurasthenia, helplessness and malnutrition pitiable in the extreme.

After the fall, the ovarian pains increased, and cephalalgia and rachialgia supervened. Tenderness over both ovaries and all along the spine gradually increased, until the patient became so hyperæsthetic that taking care of her was a problem indeed. She began then, also, to suffer from dysuria at the menstruations. At first the various hyperæsthesiæ were noticeable at the menstrual epoch. Gradually they became of longer duration, and ultimately were upon her continuously.

Soon after the fall before alluded to, local treatment was begun for a retroflexed uterus, and from that day to this she has had various treatment, but all of no avail. Pessaries of all designs for retroflexion were used (she once told me that she had used "ring pessaries varying in size all the way from a lady's finger-ring up to the equator.") A year ago, division of the cervix was resorted to, to correct the retroflexion. This was followed by the use of cervical dilators for a time, when the flexion was declared cured. But the dysmenorrhœa continued to increase in severity each month, till, in November last, she began to shed dysmenorrhœal membranes, in shreds and patches, which continued till June last.

In May, her dysmenorrhœal sufferings seemed to have reached the limit compatible with human endurance. At this time, the pains began three or four days before the appearance of the flow. Their manifestation was akin to the pains of Trousseau's "epileptiform neuralgia." Preceded for a brief time by the usual premonitory mutterings of warning in the abdomen, head, and spine, which rapidly increased in severity, till suddenly a violent flexion of the trunk on the thighs occurred, with her head thrown between the feet or legs, to be followed by as sudden a spasmodic movement throwing her into other various distortions, the periodic sufferings began. The periods of jactitation varied in length, and were accompanied with a general and exquisite hyperæsthesia; a cardiac asthenia and its accompanying pallor; an agonizingly haggard countenance; a supersensitiveness to sounds and sight; an anorexia and dysuria which seemed quite enough to fill the measure of her sufferings. These sufferings continued unchecked for periods varying from a few hours to three or four days before the flow, and for two or three days during the flow, when they gradually subsided and eventually disappeared. The physical prostration of each period was so great that she failed to accumulate any strength before the next period, and in this way her nutrition became depraved so that she was emaciated like one about to die of tuberculosis. At the rate at which she was progressing last spring, but a few more months of life apparently remained to her.

In May last, she began as usual to have severe pains and jactitation, which lasted about six hours, when she had two convulsions in rapid succession. After the last one she ceased breathing, and artificial respiration was resorted to for a period of four hours before she breathed spontaneously. After four days of great suffering, without any more convulsions, the flow supervened, and continued four or five days, with intense suffering.

After this period she was very feeble. She seemed to gain no strength, and when the June menstruation came on, she was about as feeble as she could be and endure much suffering. At this time, Dr. Emma N. Nichols, of Chicago, was with the patient, and through her skill and tireless watchfulness, the pains were held greatly in check comparatively by opiates and bromides. She remained in the room with the patient night and day till her sufferings were past. The flow came on the fourth day, and the patient's extreme asthenia was marked by very feeble heart action. Nothing else especially marked this period.

In two weeks after the cessation of the flow, the patient was brought to Chicago, June 16, 1882, a distance of 250 miles, to undergo normal ovariotomy.

It should be mentioned, *en passant*, that in the past everything in the way of remedies had been used to control the sufferings of the patient. Opiates; bromides; the solanaceæ; chloroform; ether; heat; cold; electricity; had all been tried, but to no avail.

At this point, let me pass in review, 1st, the condition of the patient on her arrival in Chicago; 2nd, the objections to öophorectomy; 3rd, the justifiability of the operation in her case.

Here was a patient presenting, objectively, two conspicuous conditions, viz: extreme dysmenorrhœa and chronic cerebral meningitis. The two together were rapidly killing her. The dysmenorrhœa was exaggerating, and, to all appearance, increasing the meningitis. So long as menstruation was performed, there could be no hope of curing or holding in abeyance the effects of the intracranial disorder. The disturbance of the nervous centers incident to menstruation always produced fresh meningeal congestion, which, superadded to the chronic meningeal trouble already existing, served only to push the patient helplessly along to the point beyond which recovery would become impossible. Till menstruation was stopped, attempts at checking the meningitis would avail nothing, and as the menopause was twenty years distant, its artificial production became debatable. Unless some cogent reasons exist why this step should not be taken, would I have been justified in withholding oöphorectomy and allowing this patient to die? General objections have been made to Battey's operation, and they are the following:

1st. Oöphorectomy is unjustifiable, because it unsexes a woman, *i. e.*, it prevents her conceiving and bearing children. This objection is needless, because the condition of my patient had already accomplished this for her very fully. Furthermore, she never would become pregnant till able to be up and around, sufficiently at least to marry, and she could never do that as long as she menstruated and grew feebler every month.

2nd. It has been said that the operation destroys sexual desire, and hence is unjustifiable. Whosoever urges this objection speaks whereof he knows not, since those patients who have given testimony hereon almost unanimously aver that oöphorectomy does *not* destroy sexual desire. Quite to the contrary many women have said that the operation has been followed by increased sexual desire. In some cases, after the operation, the patients have been described as "being actually aggressive in their demeanor." I have seen oöpharectomy performed on seven patients and each one of them was bedridden, suffering, and helpless to an extent pitable in the extreme. It is quite impossible to understand how, in a single one of these cases such an argument against the operation could be seriously mentioned.

3rd. Again, it has been urged that useless operations will be performed. This must be granted, but it must be granted in regard to other surgical operations also. I have known a case of lithotomy where no stone existed; of a thoracentecis where no fluid existed; yet these failures were no argument against these operations.

4th. It has been said that oöphorectomy tends to masculinize a woman, as castration in the male tends to make him effiminate. Here the parity of the reasoning is lost. For if castration makes a man effeminate it *ought* to make awoman *more* effeminate. Herein the objector speaks also whereof he knows not, for the testimony of women thus operated on is wholly against any tendency to masculinity.

The operation performed under the carbolic spray, needs but a brief description. I was assisted by Dr. Davenport of the hospital staff, Dr. Nichols, and Prof. Parkes. The abdominal method was followed. The ovaries were easily secured and ligatures were applied as near to the uterus as possible, in order to remove as much of the tubes as I could. The spermatic arteries were also tied. The left ovary was slightly adherent, but was easily separated from its attachments. After closing the wound in the usual manner, and antiseptic dressing being carefully applied, the patient was put to bed. Reaction followed in three hours satisfactorily.

Recovery from this time was tedious and trying in the extreme. The wound was nearly three months in healing completely. A bed-sore developed which was two months in disappearing. The general innutrition of the patient constituted the most annoying element in retarding recovery.

When the next menstrual period was due, the patient developed the usual prodromata, but anodynes controlled the sufferings greatly. A great diminution of sufferings and exhaustion was apparent.

The second period presented the usual sufferings to begin with, but lasted a few hours only. It was followed by many days of hallucinations and mental vagaries, accounted for only on the ground of cerebral innutrition. Generous feeding and very free stimulation slowly relieved her of this condition.

The third period was characterized by the usual prelimina[illegible] sufferings, which very readily yielded to remedies, and was characterized by a brief after-period of depression.

Shortly afterward, the patient was taken home, and the fourth and last period after the operation was the briefest in suffering

Thus it would seem that slowly the habit of the nervous syste[illegible] in a bad direction is being destroyed. Soon the patient will b[illegible] free from the monthly exacerbations of suffering, and she will los[illegible] trace of the menstrual function. Other oöphorectomy patients experience a similar reluctant abandoning of periodic suffering. At no time since the operation has the patient had anything answering to a menstrual flow. If ovulation produce menstruation, then I have no fear of the latter ever supervening again, because the spermatic arteries were obliterated by ligature, and any minute piece of remaining stroma of ovary will become atrophic and unable to furnish ova. Many patients frequently menstruate after this operation and after double ovariotomy. If the ovaries be wholly removed, of course no ovum is extruded in such a menstruation. For the explanation of this phenomenon it has been suggested that the menstrual flow comes on purely from habit. The latest explanation offered for menstruation after the e moval of both ovaries is offered by Lawson Tait, I believe.

According to this gentleman, the fallopian tubes preside over menstruation. Ovulation has nothing to do with it. Hence, the complete removal of the tube is necessary to stop menstruation. This is a novel view, which, if true, offers the unfound explanation for ovulation occurring independent of menstruation, and hence makes clear the phenomenon of impregnation without menstruation, *e. g.*, during lactation.

Menstruation after oöphorectomy, so far as present observation extends, usually ceases after a few recurrences, and the patient, unreminded of its presence, soon loses trace of the time in the month when she ought to be unwell.

Oöphorectomy has been proposed for other conditions than dysmenorrhœa, as, 1st, uterine myomas accompanied by hæmorrhages that threaten the extinction of life; 2d, insanity pointing clearly to an ovarian origin; 3d, recurrent hematocele; 4, pyo- and hydro-salpinx, with pelvic peritonitis; 5th, hystero-epilepsy; 6th, irreducible hernia with inflammation; 7th, intumescence of the ovary, chronic oöphoritis, perioöphoritis, and commencing cystic degeneration; 8, atresia of the uterine canal and vagina sufficient to retain the menstrual flow.

Many cases of the successful checking of hæmorrhages from uterine fibrous tumors by oöphorectomy are now recorded. The risk to life is infinitely less in removing the ovaries in these cases than in removing the tumors. When successful, the cessation of the loss of blood quickly follows castration, and atrophy of the myoma results. The largest statistics given by one writer that I can now refer to are furnished by Dr. W. Wiedow on the subject of "castration for uterine myo-fibromata," in the *Centralblatt für Gynäkologie*, February 11, 1882. Twenty-one cases of bleeding fibroids are reported where Battey's operation was performed. Out of this number, three died from the operation. Recovery, with menopause produced by the operation, and diminution of the growth, followed in sixteen cases. One patient of this number menstruated nine times, and then recovered; four menstruated from one to four times and then recovered. One patient ceased menstruating three months, and then had a hæmorrhage, after which enucleation was performed and recovery followed. In the last case, menstruation continued five months, when a hæmorrhage supervened; afterward degeneration of the tumor followed, and death closed the scene in nine months. Thus it will be seen that seventeen out of twenty-one cases recovered. And when we recall the almost uniformly fatal result of severely bleeding uterine myo-fibromata, as all of these twenty-one cases were, we see that oöphorectomy offers what no other procedure can ever produce.

A few cases are recorded where insane women have been cured by the removal of their ovaries. This has led to the sweeping supposition that all insane women should be castrated. It is evident from *a priori* reasoning that that would not be sound treatment. Dr. Putzel, the pathologist of the New York Female Insane Asylum, failed to find any uterine or ovarian diseases in over one hundred autopsies made on inmates dying at that institution, and among those very one hundred patients there were doubtless many who would have been cured by oöphorectomy. After studying carefully the histories of all the cases of insanity cured by the removal of the ovaries, I am convinced that no insane woman should be operated upon whose earlier history does not show incontestably clearly a relation between the ovarian function and her malady. In many such cases, healthy ovaries will be removed, but the exciting cause of the insanity will go with them.

Recurrent hematoceles, with their resultant threatenings to life, may be regarded as a justifiable indication for oöphorectomy. The conditions permitting these hæmorrhages, once manifesting their presence, ought to be regarded with apprehension. The fact of a second hæmorrhage in any patient would lead me to debate the propriety of removing her ovaries. An easy recovery by absorption after the first occurrence of an hematocele—not an uncommon thing—is no assurance against inflammation or sepsis after subsequent attacks. To guard against these results, the now comparatively safe operation of castration is considered consonant with good surgical judgment.

Pyo-salpingitis and hydro-salpingitis are considered of sufficient gravity to warrant the removal of the ovaries. Results of operations therefor are both surprising and gratifying. Chronic ovaritis and chronic salpingitis, with recurrent attacks of pelvic peritonitis, produce so much danger to life as to warrant this operative procedure. Patients thus afflicted are liable to become opiophagists through attempts to relieve sufferings.

Hystero-epilepsy of a clearly ovarian origin ought to make castration a debatable procedure. Many recorded cases show the wisdom of removing the ovaries in this malady. But one precaution which should control the judgment in operating in hystero epilepsy need be emphasized, and that is the incontestable relation of cause and effect between the abnormal condition of the ovaries and the manifestations of this disease. Without this relation, the wisdom of operating becomes questionable.

The two other causes enumerated above so clearly demand oöphorectomy that amplification is unnecessary.

1634 Michigan Avenue, Nov. 1, 1882.

---

BIBLIOGRAPHY.

1. Prof. Alfred Hegar. *Die Castration der Frauen vom. Phys. und Chirurg. Standpunkte aus.* Leipzig, 1878.

2. Battey. Normal Ovariotomy.—*Atlanta Med. and Surg. Jour.*, 1872, Vol. X, No. 6.

3. Prof. D. W. Yandell. Battey's Operation.—*American Practitioner*, Oct. 1875.

4. Prof. Börner. *Der anticipérte Climax durch Extirpation der Ovarien, etc.—Deutsche Medic. Wochenshrift.* Berlin, 1876.

5. H. Maruh. *Ueber einseitige Castration der Frauen. Griefswald*, 1878.

6. Prof. Wm. Goodell. On Normal Ovariotomy for some Menstrual Disorders.—*Trans. Med. Soc. Penn.*, 1879.

7. A. Lutaud. *Étude critique sur l'ovariotomie normale.* Paris, 1879.

8. W. Greve. *Beitrag zur Castration der Frauen.* Würzburg, 1879.

9. J. Marion Sims. Remarks on Battey's Operation.—*Brit. Med. Jour.*, 1877.

10. J. Matthews Duncan. Clin. Lect. on Diseases of Women. 1883

11. M. Danillo. Diseases of Sexual Organs in Insane Women.—*Der Arzt.* No. 10, 1882.

12. M. D. Moon. Normal Ovariotomy for Uterine Fibroids.—*Amer. Jour. Obst.*, N. Y., XIII, p. 793. 1880.

13. J. West. Normal Ovariotomy for Ovaralgia with Epileptiform Convulsions.—*Md. Med. Jour.*, Vol. VI., p. 315. 1880.

